# A Critical Appraisal of the Evidence Supporting Consumer Motivations for Alternative Proteins

**DOI:** 10.3390/foods10010024

**Published:** 2020-12-23

**Authors:** Rachel Tso, Amanda JiaYing Lim, Ciarán G. Forde

**Affiliations:** 1 Clinical Nutrition Research Centre (CNRC), Singapore Institute for Food and Biotechnology Innovation (SIFBI), Agency for Science, Technology and Research (A*STAR), Singapore 117599, Singapore; rachel_tso@sifbi.a-star.edu.sg (R.T.); Amanda_Lim@sifbi.a-star.edu.sg (A.J.L.); 2 Department of Physiology, Yong Loo Lin School of Medicine, National University of Singapore, Singapore 117593, Singapore

**Keywords:** meat, plant-based meat, consumer behaviour, health, sensory, sustainable, animal welfare, food choice, acceptance

## Abstract

Alternative proteins are receiving increased global attention. This burgeoning interest in plants (especially plant-based meat alternatives), insects, algae, and cultured meat has been attributed to their reported health benefits, lower environmental impact and improved animal welfare compared to conventional animal-based meat. Food producers and the media are promoting acceptance of these products, claiming superior nutritional, environmental and ethical credentials and a desirable novel sensory experience. However, the evidence supporting these claims remains unclear. In this review, we summarise the main evidence underlying the nutritional, sensorial, economical, ethical, and environmental reasons reported for the rise in consumer demand for alternative proteins. We found many of these reasons to lack a strong evidence base. For instance, evidence is emerging for the nutritional benefits of plant-based meat alternatives, but present claims are largely based on established evidence for plant-based diets. Significant research gaps remain, especially longitudinal evidence on the sustained effects of replacing conventional animal-based proteins with alternative sources. For many alternative proteins, challenges exist in achieving desirable sensory properties akin to animal-based meat to promote their acceptance by consumers. Overall, fundamental shifts in the food system are required to create a culture in which healthful and sustainable food choices are the norm.

## 1. Introduction

Sales of meat-free foods increased by 40% from 2014 to 2019 in the United Kingdom [1], while sales of Impossible Foods alone increased by more than six times in Singapore, Hong Kong, and Macau in 2019 [2]. This surge in demand for animal-free alternative proteins has been attributed to increased consumer interest in health alongside rising concern for the environment and animal welfare [3]. Another reason that is offered for the move to find new protein alternatives is the projected shortfall in global protein availability to meet the needs of a rising population [4,5]. In 2020, the COVID-19 and African Swine flu pandemics have been linked to a surging demand for plant-based meat alternatives (PBMAs) due to concerns around food security and risk of disease from animal-based foods [6]. According to Nielsen data, PBMA sales more than doubled in the United States in April 2020 during the COVID-19 pandemic compared with the previous year [7]. Widespread media reports have promoted acceptance of these trends, with much less attention given to the available evidence to support many of the reasons that are claimed to be driving consumer behaviour.

Plant-based protein producers are targeting the rising number of flexitarians that are attempting to reduce their meat consumption [8,9]. Reducing rather than removing meat is seen as a more manageable dietary change that is more acceptable to consumers [10,11]. However, despite more people reporting to reduce or limit their meat intake [1], overall and per capita meat consumption has been steadily increasing globally over the past three decades [12]. This has been attributed to declining meat prices, advances in meat production technology, globalisation of food systems and increased affluence in low-to middle-income countries such as China and India [12,13,14,15]. The result is a wider accessibility to a larger variety of protein sources for more people who can afford them. With more options to choose from, consumers are now in a position to consider health-promoting effects, price, taste, environmental concerns, and animal welfare as important factors when making food choices [1,16]. However, the relative influence of each factor, and the associated evidence to support and verify claims made for each of these consumer drivers remains unclear.

Research into the nutritional, environmental, and sensory aspects that affect consumer acceptance of newer alternative protein sources is in its infancy. Despite the significant increase in global media interest in alternative proteins, the evidence supporting some of the key drivers of consumption is mixed, and consumer acceptance of new plant- and insect-based alternative proteins is not universal. This may also be due to cultural differences as there is widespread acceptance of PBMAs and cultured meats in China and India [13], whereas consumers in the United States and Europe are reported to have more polarised views [13,17,18].

The current review begins by (i) defining the different categories of established and emerging alternative protein sources, and will (ii) summarise the main reasons reported for the recent rise in demand for alternative proteins, (iii) and within each, weigh the strength of available evidence for each factor reported to be driving consumer demand for alternative proteins. Finally, we (iv) highlight some of the research gaps in our current understanding of what motivates consumers to make the sustained switch to alternative proteins as part of a dietary change which is both healthful and sustainable.

## 2. What Are ‘Alternative Proteins’?

Conventional proteins comprise animal-based protein sources such as beef, fish, chicken, and dairy, whereas alternative proteins are those from non-animal-based sources. Table 1 summarises the four main alternative protein categories that will be discussed throughout this paper. Plant-based foods include vegetal sources such as cereals and legumes, and are traditional parts of diets around the world. By comparison, many of the new PBMAs incorporate purified plant proteins into processed products that mimic the appearance and experience of eating animal meat. Insects are widely consumed in parts of Africa, South America, and Asia [19], and are traditionally cooked before consumption [20]. Insect consumption is less prevalent in Western countries though recent advances in processing has led to their increased use as ingredients—which has greater acceptability among consumers and is gaining popularity in more familiar foods such as biscuits and bread [21]. Edible algae such as seaweed, *Spirulina,* and *Chlorella* are traditionally a part of diets in Asian countries [22], with rising global demand in recent years [23]. Algae and seaweed are typically processed or cooked before consumption or their components extracted and incorporated into health food products for their purported functional benefits [24]. Cultured meat applies laboratory tissue culture techniques to produce ‘lab-grown meats’, and innovation in their production has rapidly increased since the first proof of concept in 2013, with the advantage that cultured animal cells can be used to produce ‘meat’ in a laboratory, without the associated animal cruelty or environmental impact [25]. Cultured meat is not yet commercially viable or available due to the associated production costs, and current consumer reticence to consume ‘lab-grown meat’ [26].

## 3. Summary of Evidence on the Motivations to Consume Alternative Proteins 

This section provides a summary of the available evidence for several key factors reported to influence consumer motivations for consuming alternative proteins. Consumer acceptance of novel foods is complex, and long-term changes to dietary behaviour go beyond enhanced product sensory appeal and liking. Evidence shows that product acceptance can be strongly influenced by psychological factors including situational and emotional factors, where specific situations can promote a positive arousal and impact product acceptance for alternative protein such as insect-based foods [29,30]. In addition, consumers’ food neophobia and perception of disgust have been shown to affect acceptance of alternative proteins [31,32], and could be barriers to consumer acceptance of alternative proteins such as insects and cultured meats in the future [21]. The current review focuses primarily on the health, environmental, sensory, safety, and animal welfare concerns as the main reasons reported to motivate consumers to opt for alternative proteins, with the perception that these options are healthier [22,33], better for the environment [34], and less cruel to animals [35]. Sensory characteristics have not been widely studied across different protein alternatives, in particular the new PBMAs, but are important factors in motivating consumer acceptance of these novel products [36]. Similarly, although often less widely reported, is the rising consumer mistrust of food producers and the economic factors that are also influential in consumers’ decision-making around alternative proteins. The evidence for these factors will be explored in more depth in the following section.

### 3.1. Nutritional Quality of Conventional and Alternative Proteins

#### 3.1.1. Nutritional Concerns of Consumers about Meat Consumption

Consumers are concerned about the health impact of meat consumption as a result of several high-profile reports. The EAT-Lancet report included the findings of several meta-analyses, highlighting an association between higher processed and unprocessed red meat consumption with increased risk of total mortality [37]. The International Agency of Research on Cancer classifies processed meat as “carcinogenic”, and red meat as “probably carcinogenic” [38]. Consumers are advised to lower their red meat consumption to reduce intakes of harmful nutrients such as cholesterol and saturated fat, though the claims about negative health effects arising from red meat consumption may not be supported by strong scientific evidence [39]. This has led to the belief that substituting animal meat for PMBAs is better for health [40]. However, the evidence for this is largely drawn from comparisons of red meat to traditional vegetarian diets, and many reports fail to state the specific health effects of substituting meat with PBMAs [41,42]. As a result, it is unclear whether health claims across different alternative protein sources are supported by empirical evidence. There is strong evidence for the health benefits of consuming plant-based diets in place of a diet high in animal products [43,44], but research is sparse for the health impact associated with the sustained replacement of meat with PBMAs, algae or insect proteins, and there are currently no studies for cultured meat (Table 2). The putative health benefits of plant-based diets based on these newer PBMAs are rarely supported by empirical evidence from randomised controlled trials when making claims about the health effects of PBMAs (Table 2). Many marketing and media sources rely on general and well-established health benefits of vegetable-based diets compared to diets high in animal products when making claims about the health properties of PBMAs and the many newer, formulated alternative protein products, which may be misleading.

#### 3.1.2. Comparing the Nutrient Densities of Conventional and Alternative Proteins 

There are currently objective, standardised criteria for comparing individual macronutrients such as fat, carbohydrate, and protein [49] as well as indices including the Nutrient Rich Food index which compare the nutritional quality of foods based on several nutrients [50,51]. However, limited comparisons have been completed to date between traditional animal and alternative proteins in terms of their nutrient contents in the form in which they are normally consumed. It is more appropriate to compare conventional and alternative protein nutrient contents accounting for typical preparation methods and seasonings, rather than base comparisons for individual nutrients in the raw form, such as total protein content or protein quality alone. For example, a ‘raw’ plant-based protein may appear nutrient rich but if it requires preparation with oils and added salt to enhance sensory appeal before consumption, it is better to account for these additions when comparing their nutrient contribution to a diet. A summary of the nutritional contents of processed fish sources, ‘mock meats’, plant-based proteins, PBMAs, algae, insects, and non-dairy products in comparison to their animal protein equivalents is presented in Table 3, Table 4, Table 5 and Table 6. Using this approach, we compared the total nutritional content of plant-based and meat-based proteins across several categories: Conventional meat versus processed meats and mock meat (Table 3); beef versus legumes, algae and insects (Table 4); beef versus PBMAs (Table 5); and dairy versus dairy alternatives (Table 6). This enabled direct comparisons of nutrients across variants within each category. For example, for fish protein (Table 3), tempeh (Table 4) and insect burger patties (Table 5), each provide over 20 g protein per 100 g serving, yet the high sodium content of insect patties and high fat content of tempeh may ameliorate their nutritional benefits, and reduce the health appeal of specific protein alternatives when recommended over fish. Similarly, compared to burger patties, alternative protein burgers generally had similar calories, no cholesterol, lower fat and higher fibre, but had lower protein and often a 2–3 fold increase in sodium (Table 5). Bread containing cricket flour has been proposed as an alternative to traditional wheat breads, and has the highest protein content overall among alternative protein sources (Table 4). However, cricket bread contains approximately 10% more calories due to the high unsaturated fat content of the added insects, which is beneficial in small amounts for cardiovascular health, but may increase energy intake and contribute to weight gain if widely consumed over time. Compared to black beans (Table 4), black bean burger patties (Table 5) had similar protein content but significantly more calories, fat, sugar, and sodium, and reduced fibre and iron. This was due to the addition of preservatives and other ingredients to this plant-based burger to enhance its function and palatability.

In addition to the nutrient composition of each protein alternative, the food format (i.e., fish versus fishballs), cooking method and condiment use associated with their consumption can also significantly impact their nutrient content, as seen when comparing the different preparation styles of fish (Table 3) and tofu (Table 4). In this regard, some alternative proteins may become a vehicle for the consumption of unhealthy cooking practices, flavourings, or condiments. Much of the focus to date has been on shifting consumers from animal- to plant-based versions of fast foods or ‘junk foods’, rather than an increase in nutrient-dense vegetable-based foods. Sales data demonstrate that burgers and sausages are the top-selling formats of PBMAs [57,58]. Whereas the protein content of PBMAs is generally not a concern (up to 20 g per serving; Table 5), regular consumption of fast food-style meals is frequently associated with increased intake of calories, sodium, and fat, depending on other foods consumed during the meal. Consumers may therefore be motivated to consume PBMAs due to health marketing largely based on established evidence related to vegetarian diets (Table 2), while not considering the nutritional impact of an increased fast food intake. The risk is that promoting the health benefits of alternative proteins (especially PBMAs) over animal proteins and only offering them in formats such as convenience or fast foods may ‘license’ consumers to increase their intake of a wider variety of energy-dense, nutrient-poor foods, while believing they are eating healthily. Previous research has shown that health labelling can sometimes result in increased calorie intake, where consumers often feel entitled to increase their intake due to the assumption that the product is healthy [59,60]. With current meat intakes rising alongside the growth in popularity of PBMAs [1,2,12], this trend could encourage consumers to increase their intake of energy-dense, nutrient-poor foods that are often higher in salt, sugar, fat, and energy, where greater consumption is associated with an increased prevalence of diet-related chronic diseases [61]. Several of the more recent PBMAs contain novel ingredients, where the long-term impact of increased consumption is not well understood. For example, Impossible Foods contain soybean haem, and higher haem iron intake has been associated with increased risk of type 2 diabetes [62]. Studies on the longer term health effects of consuming PBMAs are currently lacking. In addition to the direct comparison of substituting animal- with plant-based protein sources, there is less of a focus on the nutritional impact of simply consuming less of both. Nutritious diets could also simply mean consuming less animal products and shifting the remaining consumption towards more nutrient-dense plant-based foods rather than simply plant-based protein. This includes increased consumption of vegetables and legumes, without necessarily increasing intakes of novel, highly-processed alternative proteins.

Replacing traditional animal dairy products with non-dairy milks and vegan eggs could also have nutritional consequences, as these products tend to have lower levels of protein and minerals including calcium, unless fortified (Table 6), while many nut milk dairy alternatives have higher levels of added sugar [63]. If consumed in isolation, vegan eggs contain no iron, calcium or potassium, and would only have small amounts from milk if added in a mixed dish such as scrambled eggs. Those choosing a vegetarian, vegan, or flexitarian diet will therefore need to obtain these nutrients from other foods in their diet. Similarly, many alternative proteins contain high quantities of added salt, flavourings and other industrialised ingredients that frequently require preparation through frying in oil or consumption with high fat condiments—for instance, PBMA burger patties, sausages, and nuggets.

**Table 6:** Comparison of dairy and dairy alternatives.

	Dairy Milk (Whole, Unfortified) ^1^		Soya Milk (Unsweetened, Unfortified) ^2^		Soya Milk (Unsweetened, Fortified with Calcium) ^1^		Almond Milk (Unsweetened, Unfortified) ^1^		Almond Milk (Unsweetened, Fortified with Calcium) ^1^		Hen Egg (Scrambled) ^1^		Vegan Egg Substitute (Scrambled) 3	
	Per Serving (240 mL)	Per 100 g	Per Serving (240 mL)	Per 100 g	Per Serving (240 mL)	Per 100 g	Per Serving (240 mL)	Per 100 g	Per Serving (240 mL)	Per 100 g	Per Serving (50 g)	Per 100 g	Per Serving (50 g)	Per 100 g
Calories (kcal)	148.84	61.00	80.52	33.00	80.52	33.00	131.76	54.00	36.60	15.00	123.15	175.93	128.70	183.85
Protein (g)	7.69	3.15	6.59	2.70	6.98	2.86	5.08	2.08	1.44	0.59	6.67	9.53	6.15	8.79
Carbohydrate (g)	11.71	4.80	2.93	1.20	4.25	1.74	3.05	1.25	1.42	0.58	1.20	1.71	1.86	2.65
Sugar (g)	12.32	5.05	2.68	1.10	1.00	0.41	1.02	0.42	0	0	0.86	1.23	0.76	1.08
Total Fat (g)	7.93	3.25	4.88	2.00	3.93	1.61	11.18	4.58	2.68	1.10	9.97	14.24	10.67	15.24
Saturated Fat (g)	4.55	1.87	0.63	0.26	0.50	0.21	1.02	0.42	0	0	2.21	3.16	0.61	0.87
Polyunsaturated Fat (g)	0.48	0.20	2.27	0.93	2.46	1.01	-	-	0.58	0.24	2.21	3.16	3.00	4.29
Monounsaturated Fat (g)	1.98	0.81	0.90	0.37	0.96	0.39	-	-	1.73	0.71	4.79	6.84	6.38	9.12
Cholesterol (mg)	24.40	10.00	0	0	0	0	0	0	0	0	207.00	295.71	1.50	2.14
Sodium (mg)	104.92	43.00	29.28	12.00	90.28	37.00	4.88	2.00	173.24	71.00	70.95	101.36	199.63	285.19
Fibre (g)	0	0	1.95	0.80	1.22	0.50	0	0	0	0	0	0	0	0
Vitamin B12 (µg)	1.10	0.45	-	-	2.71	1.11	-	-	1.54	0.63	0.58	0.83	-	-
Iron (mg)	0.07	0.03	0.98	0.40	1.12	0.46	1.02	0.42	0.85	0.35	0.84	1.20	0	0
Zinc (mg)	0.90	0.37	0.49	0.20	-	-	-	-	0.17	0.07	0.68	0.97	-	-
Calcium (mg)	275.72	113.00	24.40	10.00	302.56	124.00	7.32	3.00	480.68	197.00	40.95	58.50	16.95	24.21
Potassium (mg)	322.08	132.00	275.72	113.00	292.80	120.00	163.48	67.00	183.00	75.00	85.80	122.57	19.80	28.29
Protein Source	Casein, whey		Soy		Soy		Almonds		Almonds		Lipoproteins, ovalbumin		Mung beans	

Sources: ^1^ United States Department of Agriculture [52], ^2^ Health Promotion Board [53], ^3^ Internet source [64]. Nutritional information of all food items (as prepared and ready-to-eat) was calculated by adding fat (oil or butter) and sodium (salt or soya sauce) to raw ingredients. Dashes denote unavailable values.

Preliminary findings from the first extended consumption trials comparing alternative protein consumption to meat consumption suggest that simple substitution of animal- for plant-based proteins may have side effects beyond protein balance. A recent 12-week randomised controlled trial found increased markers of poorer bone health in the plant-based arm [65]. A slightly longer 16-week crossover randomised trial comparing PBMAs (Beyond Meat products) with animal-based meat found the former to improve cardiovascular risk factors, including trimethylamine N-oxide (TMAO), relative to eating meat [66]. Thus, initial findings are mixed, and suggest the need for further longitudinal studies and randomised controlled trials to explore and validate the impact of alternative proteins on health and body composition, beyond protein intake alone. In the absence of robust evidence supporting the health claims made for many newer alternative proteins, there is likely a “health halo” surrounding them, particularly for PBMAs, where health factors from traditional vegetarian diets are possibly being conflated with sustainability and environmental reasons to drive consumer opinion.

### 3.2. Consumer Attitudes, Sensory Appeal, Novelty, and Price of Alternative Proteins

#### 3.2.1. Do Alternative Proteins Have a Higher Sensory Acceptance than Animal Meat?

Technological advancements in processing and flavouring have meant many alternative protein products are now more widely accepted by consumers, compared to the original meat substitutes such as mock meats that were first introduced [67]. Products such as the Impossible Burger and Beyond Burger claim to replicate the appearance, texture and taste of beef burgers, and can account for the ‘bleed’ associated with cooking [68]. These properties have been achieved through ingredient innovations that can mimic meat flavour and texture using combinations of plant-based proteins and fat [69]. However, when compared side by side, consumer liking of commercially available plant- and insect-based burgers tend to be significantly lower than their appreciation for meat-based burgers [70]. This suggests that there remain challenges in terms of achieving an acceptable sensory profile that adequately matches that of cooked animal meat. These challenges differ across the various types of alternative protein sources for the meat sensory properties they are trying to replicate.

Consumer surveys highlight the belief that products made from alternative proteins are expected to have an inferior taste, texture, and sensory appeal when compared to conventional meat products [71]. These expectations are often formed by product appearance, and many alternative protein products do not resemble the appearance of traditional animal-based foods during cooking or when consumed. For insect burgers, remnants of the original insect may also deter consumers [72,73]. Incorporating algal proteins such as *Spirulina* and *Chlorella* can also pose a challenge as they can impart a distinct blue-green or green colouration [74]. These differences have been shown to impact consumer acceptance of a ‘chicken rotti’ enriched with *Spirulina* and *Chlorella* when it was rated lowest by consumers when compared to the same dish enriched with soy beans, lentils, or broad beans [75].

Many alternative proteins struggle to deliver meat-like textures [69,76,77], making it challenging to replicate the complex sensory profile and dynamic temporal changes that occur during consumption of animal meat products [78]. Even a partial (5%) replacement of meat with insects negatively affected the structural and physical stability of sausages, making them crumbly and producing inferior textural properties compared to conventional meat sausages [79]. Initial reports also confirm that the first cultured beef burgers were described as “dry”, “lean”, and “not juicy”, compared to traditional animal meat patties [80]. Research is ongoing to cultivate fat cells in vitro in combination with these cultured muscle tissues, to recreate a blend of proteins and fats that can provide a juicier texture [69].

The degradation of animal muscle and fat cells during cooking releases certain flavours and aromas [69], which can be challenging to recreate among alternative proteins. Traditional plant-based proteins release volatile compounds during preparation due to oxidation of unsaturated fatty acids, resulting in off-flavours and odours. Soy protein has a ‘beany’ or ‘grassy’ flavour due to lipooxygenases, whereas saponins and isoflavone compounds cause a bitter and astringent off-flavour [77]. In general, algal proteins have a fishy taste [81,82], and some, for example *Spirulina*, have strong ‘earthy’ and ‘musty’ flavours that are undesirable, making them difficult to incorporate into a wide range of foods [83,84]. Meat products supplemented with *Spirulina* and *Chlorella* have received low consumer acceptance as they tasted bitter due to the presence of high concentrations of amino acids [85]. The addition of spices, seasonings and flavouring agents has been shown to mask off-flavours in many alternative proteins, especially PBMAs [76]. However, the margin for error in designing such foods is larger because adding flavours may quickly polarise consumers who have developed clear expectations of what meat products ‘should’ taste like. For example, Chiang et al. found that the addition of beef bone extract that had undergone enzymatic hydrolysis and the Maillard reaction could be used to improve the ‘meaty’ taste and aroma in PBMAs [86]. However, these PBMAs can no longer be labelled “meat free”, “vegan” or “vegetarian”, and in some cases the addition of a 40% beef bone extract resulted in the PBMA having a ‘burnt’ appearance and bitter side taste that produced a poor sensory quality [86,87].

Off-flavours and changes in colouration can reduce the sensory acceptance of alternative proteins when consumers do not expect these attributes to be present. Product acceptance and repeated consumption are driven by a product’s sensory appeal [17]. A sustained move towards consuming alternative proteins requires an equivalent or superior sensory profile to that of meat [88].

In addition to the sensory challenges of alternative proteins, product label information has been shown to bias sensory properties and consumers’ perceptions. Insect burgers were least liked and rated the lowest for quality and nutritional value, as consumers were unfamiliar with consuming insects and associate insects with disgust, being exotic, primitive, and of lower nutritional value [89]. However, when informed of the environmental benefits and assured of their safety, the liking and perceived nutrient value of insects increased [70]. Whether this phenomenon would transfer to food choice and intake behaviour beyond a laboratory to sustained choice and dietary patterns in the future remains to be seen. Research to date has focused on acute measures of consumer acceptance and choice, and there remains a shortage of long-term studies to map consumer acceptance of novel alternative protein foods over time.

#### 3.2.2. Is Novelty Driving the Acceptance of Plant-Based Meat Alternatives?

Beyond enhancing the sensory appeal of alternative proteins, there is a need to better understand the psychological, situational, and emotional factors that are known to influence consumer acceptance for novel food products in general. These factors are part of the multidisciplinary factors highlighted by Köster in a comprehensive overview of the main determinants known to influence consumers’ food choice [29]. Evidence suggests that emotional responses such as disgust are likely to vary across different alternative protein sources, where insect-based or cultured meat proteins are more likely to evoke disgust [10,90]. The consumption context of a product will also significantly influence its perceptual appeal, as evidenced by recent research [31,32].

The top two factors given by consumers in the United States for trying PBMAs were “liking to try new foods” (41%) and “curiosity” (30%) [91]. “Discovering new taste” was also one of the top reasons motivating consumers towards a more plant-based diet [92]. Fast food chains have quickly capitalised on this trend, and the major franchises now offer plant-based alternatives of popular meat-based burgers as menu items alongside their vegetarian options in an effort to capture the trend towards the plant-based category [93]. However, whether the initial novelty of these new plant-based fast foods will sustain a wholesale move to greater plant-based diet consumption remains to be seen. Novelty may drive consumer appeal in the short term, but a longer-term barrier to acceptance of novel alternative proteins is food neophobia, arising from consumers’ concerns associated with consuming novel or unfamiliar products [30]. Evidence suggests food neophobia is central to the rejection of insect-based and cultured meat proteins, but less is understood about the impact of neophobia on long-term plant-based protein acceptance [10,90,94]. Research has shown that ‘mere-exposure’ increases consumer familiarity and liking for novel products, and may diminish the effect of neophobia over time [95]. Sensory acceptance alone is unlikely to motivate significant long-term consumer behaviour change towards alternative proteins, and future research should consider approaches that reduce food neophobia and promote sensory appeal for alternative proteins [10].

Many food trends such as the ‘frozen yoghurt craze’ and ‘unicorn-themed’ foods were unable to sustain the hype they initially received [96,97]. In addition, rather than focusing on making all alternative protein products mimic the taste, texture, and appearance of meat products, there may also be scope to create a new category of non-meat-like, plant-based protein foods that can help sustain the initial novelty and appeal, and broaden the scope of sustainable protein sources available in the diet. Future research should focus on repeated exposure and long-term consumer acceptance of the perceptual properties of novel PBMAs to better understand whether the current trend is driven by novelty, or reflects a more meaningful shift in consumer preference towards these new products. In addition to sensory appeal, there is now a concurrent need to understand the psychological, situational, and emotional factors that influence consumer acceptance for novel alternative proteins.

#### 3.2.3. Is the Price of Alternative Proteins Driving Their Acceptance?

Consumer choice is also influenced by price, and currently many of the alternative proteins are significantly more expensive than animal-based proteins. Plant-based proteins such as soy and pea protein are cheapest at US$2 and US$5 per kg protein, respectively, whereas insect powder (US$41/kg) and cultured meat (US$300/kg) are significantly more expensive [3]. New PBMAs such as Beyond Sausages contain pea protein isolate rather than fresh peas, making them approximately 70% more expensive than conventional pork sausages [98]. A higher price may be driven by early demand for these new PBMAs, but is unlikely to be sustained, with analysts recommending that PBMA costs will need to significantly decrease if they are to be price-competitive with conventional meat in the long term [99]. The first cultured meat burger was priced at US$280,000 due to the high cost of the cell culture growth medium [100]. Currently, consumers are willing to purchase PBMAs at higher premiums [101], possibly due to the widely-held belief that healthier foods are often more expensive [102]. However consumers are less willing to pay the same premium for insect-based or cultured meat products, despite their added production costs [103]. Research shows consumers are likely to purchase cultured meat at a lower price [16], as the perception is that these cultured meat products are expected to be cheaper since they are produced in a laboratory to meet increased global demand for protein [104]. Cultured meat is currently not available on the market and research suggests that improving consumers’ understanding of how cultured meat is produced may help promote acceptance of the higher price [105].

### 3.3. Sustainability, Environmental, and Animal Welfare Concerns

#### 3.3.1. Are Consumer Concerns about Sustainability and the Environment Driving Acceptance of Alternative Proteins?

With the world’s population predicted to exceed 9 billion by 2050, there is a projected increase in demand for protein, in order to bridge what has been termed the ‘protein gap’, or shortage in global protein production to meet demands [4,5]. However, the evidence suggests that the ‘gap’ may currently be informed by estimates that are based on the overconsumption of animal protein in developed countries such as the United States and Europe [106,107]. Red meat and poultry production in the United States has seen year-on-year increases due to a rising demand for animal meat products [108]. Estimates of the global protein needs for an enlarged population are attributed to large differences in the distribution and availability of high quality protein in different regions, where countries in Europe currently consume a higher proportion of animal proteins compared to countries in Asia where plant proteins occupy a larger proportion of the protein supply [109].

Studies show that animal production is a significant contributor to climate change due to substantial land, water, and energy use, as well as considerable greenhouse gas (GHG) emissions during production [110,111]. Livestock feed production currently uses an estimated 33% of arable land, and accounts for approximately 10% of global GHG emissions [111,112]. Beef production results in an average of 50 kg of GHG and uses 164 m^2^ of land per 100 g of protein, which is estimated to be 20 to 100 times higher than other protein sources such as tofu (2.0 kg GHG, 2.2 m^2^ land), nuts (0.3 kg GHG, 7.9 m^2^ land) and peas (0.4 kg GHG, 3.4 m^2^ land) [113]. In terms of crop usage, animal production requires 2 to 7 kg of crops to produce 1 kg of poultry, pork, or beef, hence raising the question if it would be more effective to directly utilise these crops as a food source [112,114]. An increased global population coupled with rising affluence in low- to medium-income countries is estimated to increase the environmental impact of the current global food system by 50 to 90% [115]. This is now seen as a more urgent issue than any potential ‘protein gap’ [116]. Similarly, others have argued that a higher carbon footprint of nutrient-dense animal food production can be offset by their higher nutritional value in terms of protein quality, energy and essential nutrients per kg [117].

Life-cycle analyses (LCAs) have shown animal-based meat products to require 11 times more fossil energy than similar PBMAs or insect-based alternatives [118,119]. The Beyond Burger produces 90% less GHG and requires 46% less energy, 93% less land, and 9% less water for production [120], with similar reductions observed for the Impossible Burger [121]. Insect-based protein powder is at least two times more environmentally friendly than whey protein or poultry [122]. However, alternative proteins are not equivalent in their reduction of environmental impact. Despite faring better than cow’s milk in terms of pesticide use and fossil fuel depletion, almond alternatives use 93 times more water [119,123]. Purifying plant proteins is also resource-intensive as it requires use of acids and large amounts of water and energy in return for a low yield [124]. The environmental cost of processing may therefore diminish some of the environmental and sustainability credentials often espoused by advocates for a wholesale move to alternative proteins, especially plant-based ones.

Although environmental concerns are often cited as one of the main reasons driving consumer demand for alternative proteins, research has shown that only a minority of consumers are aware of and motivated by environmental concerns related to food production, in their desire to reduce meat consumption [11,125,126,127]. A recent systematic review of 34 studies found that only 13 to 26% of consumers were willing to stop or significantly reduce their meat consumption for environmental reasons, or have already changed their meat intake for environmental concerns [10,34]. Even among consumers who report prioritising “healthiness” and “environmental friendliness” of food, this does not translate into actual food choices [128]. Research from the United States and United Kingdom suggests that 720 billion tons of GHG could be cut if the entire world switched to a meatless diet [129], suggesting that consumers would need to cut meat consumption completely and switch to vegetarian diets to achieve a significant environmental improvement. Shifting away from meat consumption has clear environmental benefits, but this is rarely positioned side by side with other consumer changes that may have a greater impact. For example it is possible to achieve a greater reduction in GHG (940 billion tons) when continuing to consume animal meat, and instead adopting other measures such as wasting less food, using carbon-efficient farming, not overconsuming, and reducing animal consumption by choosing alternative proteins as a substitute on occasion [129]. Going car-free (2.4 tonnes carbon dioxide (CO_2_) equivalent saved per year) and cutting down on trans-Atlantic long-haul flights (1.6 tonnes CO_2_ equivalent saved per roundtrip) would achieve greater reductions in CO_2_ emissions than opting for a plant-based diet (0.8 tonnes CO_2_ equivalent saved per year) [130]. In this regard, taking one flight would offset the carbon savings of one or more years of veganism [131]. When 1% of the world’s population accounts for over 50% of global CO_2_ emissions through commercial aviation [132], it creates a potential conflict between the ‘virtue-signalling’ around removing animal products from diets due to environmental concerns, and continuing to live carbon-intensive lifestyles [133]. When global tourism accounts for ~8% of GHG emissions [134], the question must be asked whether it is more effective to reduce meat consumption or stop going on foreign holidays?

Finally, meat substitutes and PBMAs are rarely discussed in terms of carbon emissions associated with their production but in some cases have carbon footprints that are comparable to poultry products [135]. For example, ‘chicken-free’ Quorn contributes ~3 kg CO_2_ equivalent per kg [136], while other mycoproteins can be up to ~6 kg CO_2_ equivalent per kg, which is the same as poultry production [137]. Current estimates suggest in vitro meat would have a higher carbon footprint than poultry or pork [138]. Although cited as a significant driver of consumer motivation for alternative proteins, research suggests that consumers lack awareness of the environmental impact of food production and are often unaware of the relatively larger contribution other lifestyle behaviours such as international flights make to GHG emissions. Future research is required to understand whether an informed consumer is likely to reduce their consumption of animal products based on environmental concerns, and how these behaviours compare with related reductions in GHG emissions from other lifestyle changes.

#### 3.3.2. Are Consumer Concerns about Animal Welfare Driving Alternative Protein Consumption?

Animal welfare is often cited as a key motivator for consumers who desire to reduce or avoid the consumption of animal products, with vegetarians and vegans the most concerned about this issue [139,140]. A study of United Kingdom consumers concluded that almost 90% of respondents deemed it crucial that the meat they purchase is produced using good animal welfare, with females and older consumers the most concerned with animal cruelty [35]. However, animal welfare ranks lower than other factors such as health and novelty as a consumer driver for meat eaters to reduce their intake of meat [141]. Meat products are typically presented fresh, clean, and under sterile conditions that make it difficult for some consumers to connect eating meat with animal harm and suffering [142]. Many consumers rationalise their decision to continue consuming animal products despite concerns for animal welfare [143,144]. In addition, not all alternative protein sources are completely free of animal origins. As described previously, several meat flavour additives still rely on animal sources, and due to current technological and financial limitations, cultured meat still requires inputs of animal origin [100] and causes harm to animals [145]. Several PBMA products still contain egg or dairy ingredients, and thus cannot be labelled free of animal-based ingredients [100]. For instance, most Quorn products contain egg white as a binding agent [146], although they also do have a vegan range. Although flexitarianism is widely accepted as a modern consumer dietary trend, beyond vegan and vegetarian diets there remains limited evidence to show that concerns over animal welfare have yet translated into long-term changes in meat intake and an associated increase in alternative protein consumption, as global meat production and consumption continue to rise annually.

### 3.4. Do Consumers Have Concerns about the Safety Aspects of Alternative Proteins?

Safety, trust, and quality are central and assumed features of the food supply, and consumers should be assured that foods they consume are free of pathogens and contaminants associated with disease or death [147]. Table 7 lists several of the key safety concerns associated with alternative protein sources. Novel plant- and insect-based protein products are prepared using new production methods and bring risks such as unknown allergens and contaminants that are currently poorly understood. Due to the recent genesis of the PBMA market, there is currently a lack of long-term studies on novel PBMAs, cultured meats, and their derivatives. To date there have been a small number of studies reporting on the safety of insects and algae, which highlight that products derived from these sources may potentially contain pesticides [148], heavy metals [149,150] or allergens, or act as hosts for parasites [151]. A systematic review concluded that further studies are required to evaluate the long-term effects of insect consumption on human health [152]. Among the different alternative protein sources, consumers perceive insects to be the least safe to consume due to concerns that insects are dirty, carry diseases, and cause allergic reactions [153]. However, these perceptions are not supported by the available data, since processed and heat-treated insects are widely regarded as safe [20]. As with the proposed health benefits of PBMAs, consumer beliefs about food safety for insects are not always based on fact. Consumer safety concerns about the consumption of algal protein relate to exposure to marine heavy metals and contaminants (Table 7), though the levels identified in algal products have not been shown to be sufficient to pose a significant threat [154,155]. Algae is currently consumed mostly as a condiment or supplemental product that is unlikely to be eaten in quantities sufficient to cause adverse effects. The long-term effect of consuming cultured meats is currently unknown as these products have yet to reach market, but consumer surveys have expressed concern and uncertainty about their safety [26]. Consumers appear to have mixed perceptions about the safety of alternative proteins as knowledge in this area is still evolving with each new safety assessment conducted on these new products.

**Figure 1:**
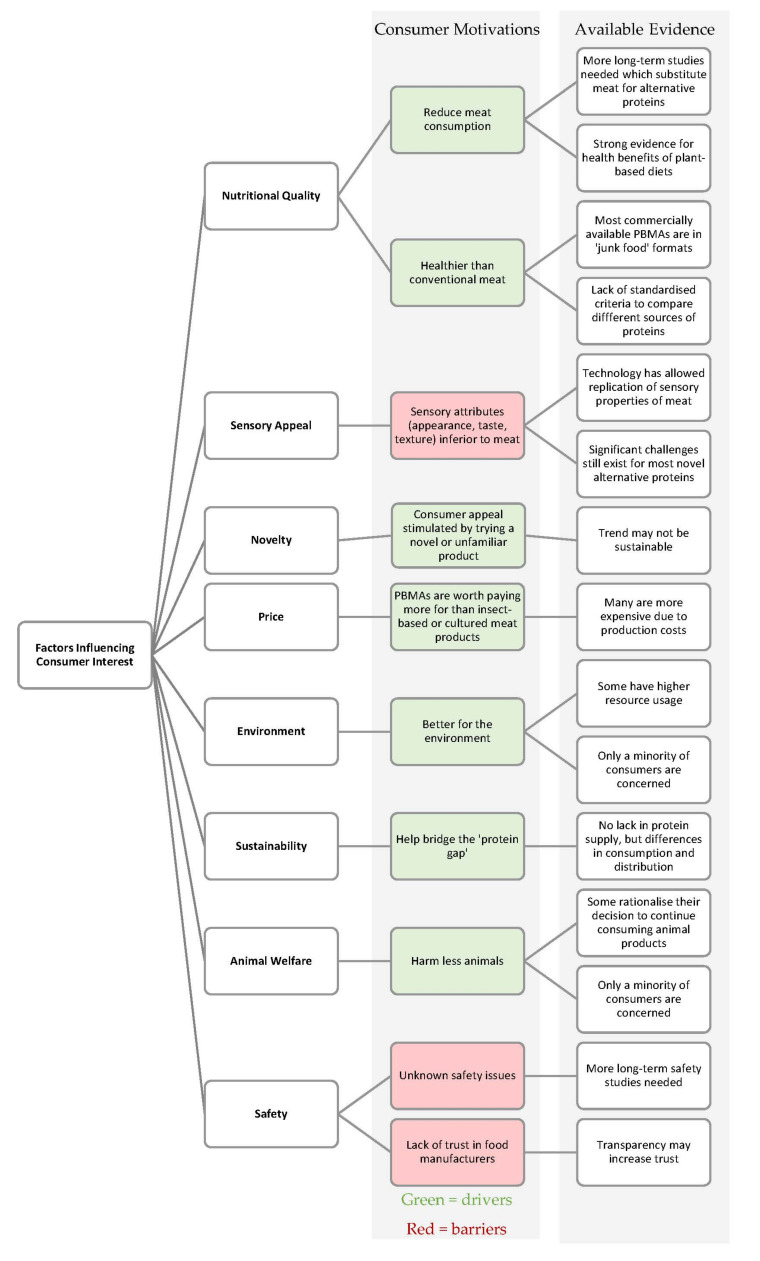
Factors influencing consumer interest in alternative proteins.

Food recalls and fraud in recent years have unfortunately eroded consumer trust in food manufacturers, with recent international controversies such as nephrotoxic melamine added to boost the protein content of infant milk formulas (2008), or horsemeat-adulterated ready meals (2013). Recent concerns about disease risk and food authenticity due to COVID-19 (2020) and the African Swine flu (2019) [6] have further influenced consumer desire for a safe and secure food supply. Consumer trust is highest for farmers and lowest for multi-national food producers and manufacturers [158]. This perception is driven by the belief that manufacturers are not honest or transparent in their food production practices. Food insecurity has stimulated an interest in alternative proteins as a means to secure the food supply chain. When trust in the food system is strong, consumers are more likely to embrace food innovations [158]. Building consumer trust is therefore a central challenge in consumer adoption and sustained consumption of novel protein sources that will require robust data and clear, non-technical communication to consumers about food production, formulation, and nutritional information. The key factors influencing consumer interest in alternative proteins discussed in this paper are summarised in Figure 1.

## 4. Gaps and Opportunities

The current review summarises some of the key reasons reported to drive the increased demand for alternative proteins, and how reported consumer drivers of acceptance are not always based on available evidence. Data to date suggest consumers are motivated mostly by health reasons when opting for alternative proteins, and less by sustainability, environmental or animal welfare concerns. There remain significant sensory challenges to improve the sensory appeal of alternative proteins, with pricing highlighted as a central concern if consumers are to make a sustained change to their dietary behaviours. Sustainability, environmental, and animal welfare concerns currently contribute less to flexitarian consumers moving towards animal-free diets. Whereas there is little doubt of the reduced environmental impact of most plant-based proteins, the evidence that environmental concerns are really motivating most consumers to reduce their animal product intake and increase their plant-based protein consumption, is currently lacking. The current projections on population growth and the GHG impact of increased global food production are likely to be stronger motivators in the future than suggestions of a global protein shortage. The current review highlights significant gaps in the available evidence, supporting many of the factors offered as drivers of consumers moving to increasing alternative protein consumption, and it remains unclear which factors will be the most influential in encouraging sustained changes to healthier and more sustainable diets in the future.

There is currently a lack of detailed longitudinal evidence on the safety, acceptance, and nutritional impact of shifting to novel alternative protein sources. Insects, algae, and cultured meat have not yet reached the mainstream consumer market and most research to date has focused on the safety and nutritional properties of traditional plant-based proteins such as soy and peas [159,160]. The emerging research on the sensory aspects of plant-based proteins highlights a number of key challenges in enhancing the sensory qualities of emerging and new alternative protein sources or for matching these qualities with animal-based proteins.

There is currently a ‘health halo’ surrounding the consumption of newer PBMAs that has been extrapolated from the extensive evidence on the benefits of plant-based versus meat-based diets. To date, there have been no long-term studies to directly compare the consumption of emerging PMBA products with traditional vegetarian or meat-based diets, resulting in a lack of evidence-based dietary guidance for the move towards them. There is therefore a pressing need for further longitudinal and controlled dietary studies to compare the nutritional impact of substituting meat and animal products with alternative protein sources over the longer-term. Research on the short- and long-term health effects of alternative proteins in different population and age groups are now needed. Studies which directly substitute animal-based proteins with plant-based proteins in double-blinded, randomised feeding trials of adequate duration are required, to investigate changes in body composition and biomarkers of nutritional adequacy such as calcium, iron, zinc, and vitamin B12. Several short-term trials are already ongoing [161] with some raising concerns about changes in bone mineral density [65] when substituting meat with plant-based foods. A recent prospective study on the EPIC-Oxford cohort found that compared to meat eaters, hip fracture risk was higher in fish eaters, vegetarians and vegans—with vegans carrying the highest risks of total, hip, leg, and vertebral fractures [162]. This was likely due to lower BMI and lower intakes of calcium and protein, though further studies are needed. More evidence is required to identify the nutrient gaps that are likely to emerge on plant-based diets that have reduced the consumption of animal products, to formulate meaningful recommendations that can provide both healthful and sustainable dietary behaviour in the future. In addition, while consumption of these novel proteins in small quantities pose little to no risk, it remains to be seen what impact a wholesale increase in intake will have on human health. Studying alternative proteins, such as the many emerging PBMA products, will however pose unique challenges due to the fluid nature of the market and rapidly changing product formulations, which make comparisons of the longitudinal impact of their consumption difficult.

Hybrid products that comprise mixtures of animal and alternative proteins offer an initial approach to encourage consumers to make dietary changes towards more sustainable protein sources, without having to compromise on sensory quality. Consumer studies have shown that unlike meat-free products, hybrid beef burgers and pork sausages were not significantly different from the full-meat alternatives in terms of sensory attributes [114]. A replacement of beef with 10% tempeh resulted in a juicier and more tender patty than the full-meat control, and was similar in terms of overall acceptability and flavour [163]. Another consumer study conducted in the United States, Spain, and Mexico found that cookies made with 15% cricket flour replacement fared similar to the control in terms of appearance, colour, and aftertaste, and consumers in Spain and Mexico significantly preferred the 15% cricket flour cookie [164]. This suggests that hybrid products have the potential to contain a blend of both conventional and alternative proteins without reducing the sensory appeal of the final product, thus supporting the consumer transition to increased alternative protein consumption. Consumer studies are required to investigate whether repeated consumption of alternative proteins, or the provision of information on their environmental and safety aspects (i.e. less direct personal benefits) will help increase acceptance of these products in the future.

In addition to environmental benefits, there are opportunities for novel protein products to be vehicles for enhanced nutrient density if they are designed appropriately to combine highly bioavailable proteins with sources of other nutrients (i.e. vitamins and minerals), while also being low in fat and high in fibre. Such foods could reduce the nutritional compromises associated with switching from largely animal- to non-animal-based diets. Nutrients such as calcium, zinc, potassium, and protein can be added to alternative milks for individuals unable to consume dairy or in cultures with a lower dairy consumption, such as Asian countries.

To achieve this ideal of nutritious alternative proteins, there is a need for standardised and objective approaches that compare the nutritional quality of alternative and conventional proteins, while also accounting for the environmental impact of their production. These metrics could be used to benchmark the nutritional content of novel alternative proteins and form a basis on which to inform consumers of the health impact of substituting conventional protein sources. Guidelines should be established on what constitutes a minimum adequacy for protein, iron, vitamin B12, zinc, and other nutrients naturally present in meat, so that alternative proteins (especially novel PBMAs) are nutritious in addition to carrying robust environmental and sustainability credentials. There is also a need to extend the current product offerings beyond fast food or ‘junk food’ formats, to ensure that the protein transition to non-animal or plant-based proteins does not inadvertently promote the sustained consumption of less healthy diets. For the majority of consumers, it may be harmful to completely abandon all meat, poultry, and dairy products, without first considering adequate replacements. Additionally, the health impact of alternative proteins should be communicated to consumers in an objective, transparent, and easily understood manner that accounts for preparation methods and the total nutritional impact of their consumption. Overall, a consumer trend to novel alternative proteins should not distract from the goal of nutrient-dense, balanced diets, with predominantly plant-based foods such as wholegrains, legumes, nuts, fruits and vegetables. This is in line with previous recommendations for diets that are both sustainable (lower in GHGs) and lower in calories. Such diets involve eating to one’s needs (‘diet quantity’), as well as consuming nutrient-dense foods and less discretionary foods (‘diet quality’) [165,166].

With a current lack of consumer trust in many food producers [158], there is an important need to encourage food companies to share necessary information about the quality and provenance of their ingredients and to be transparent about the nutritional quality and sustainability of their production methods. These messages should be non-technical and easy for consumers to understand. In a study among European consumers, a transparent system of labelling coupled with traceability mechanisms has been shown to be effective in improving attitudes towards GMO foods [167]. A similar method can be adopted for novel or non-mainstream alternative proteins. For food manufacturers, a balance must be struck between desires to protect trade secrets to maintain a competitive edge [168] and sharing sufficient detail to maintain transparency and encourage consumer trust. Increased transparency in food production will facilitate a greater acceptance of food innovations, including alternative proteins, and support the global market to move towards healthier, more sustainable food systems. At a global level, there is also a need for consistency in benchmarking the environmental impact of food production, in a way that enables naïve consumers to evaluate the true environmental implications of their food choices. Clarity on LCAs across all food sources are required to quantify and compare this aspect in a robust and objective manner. Another recommendation is for universal regulations and quality measures for alternative proteins, especially novel and non-mainstream products, to assure consumers of their safety. For instance, regulations for algae will need to account for contaminants and processing artefacts, should production scale up or specific components and isolates be refined and concentrated before their incorporation into food products. The regulatory landscape will therefore need to adapt to any potential threats that emerge from innovations in the plant-based protein space to guarantee consumer safety in the future.

Finally, the benefits of shifting away from solely animal products towards more sustainable and nutrient-dense alternative protein products will need to be framed in an appropriate way if this message is to resonate with, and be adopted by consumers. For example, labelling cultured meat as “clean meat” or “animal-free meat” was shown to evoke positive attitudes and increase their acceptance, compared to a “lab-grown meat” label [169]. Tailored communication approaches however will need to be grounded in facts and will have to be adapted for different alternative proteins and protein isolates. This remains a research challenge for the future. A study of consumer perceptions towards legumes, tofu, seitan, cultured meat, and insects failed to find a single framing approach which increased product appeal for all of the different meat alternatives [170].

An increasing world population brings an unprecedented challenge to achieve a healthy, sustainable and secure food supply for all, and will require innovative solutions, policies, and sustained changes in consumer behaviour. Fundamental changes are now needed in food production and consumer dietary behaviours to achieve and create desirable protein alternatives that extend beyond fast food formats and are affordable, appealing, nutritious, and sustainable. Food manufacturers and the media will increasingly need to play a role in promoting transparency if novel technologies are to be adopted by the public. The current review highlights a need for a stronger evidence base to support the many claims made around nutrition, sustainability, and safety before consumers will make lasting dietary shifts and adopt food innovations that support a long-term reduction in the consumption of animal products.

## 5. Conclusions

Alternative proteins have the potential to improve the sustainability of the food supply, enhance human health, and reduce harm to animals. Many plant-based foods, algae, and insects are more nutritious compared to conventional animal meats and offer new and unexplored functionalities for processing and organoleptic qualities. The rapid growth and progress in food technology now facilitates food producers to better replicate the sensory experiences of eating meat via PBMAs, and has created a new and varied market for a large number of alternative protein food options for consumers who want to reduce animal consumption to help the environment. Batch processing and exploring new substrates for food production can also support global efforts to enhance food security and diversify food production. However, consumers must be mindful of the nutritional impact of sustained consumption of many of these novel products and there is now a need for improved research and enhanced regulation to support improved health and sustainability. As technology progresses and nutritional guidelines are developed and regulated, there is an opportunity to develop a new generation of healthy and sustainable diets. There is now a need for a robust evidence base to accurately evaluate and support claims on the nutrition, sustainability, and safety benefits of alternative protein consumption, with clear and accurate communication of these benefits to better inform consumer choices and behaviours. Fundamental shifts from farm to plate are necessary to create a culture in which healthful and sustainable food choices are the norm and are easy for consumers to incorporate into their lifestyles.

## Figures and Tables

**Table 1:** Examples of alternative proteins.

	Traditional		Novel	
	Origin	Brief Definition	Origin	Brief Definition
**Plant Protein**	Traditional part of diets globally	Vegetal sources of protein that include cereals and legumes Examples: cereals include wheat, corn, rice and oats; legumes include soy and beans and products based on these, e.g., tofu, tempeh	Beyond Meat’s first product, Chicken-Free Strips, was launched in 2012 Impossible Foods launched The Impossible Burger in 2016	Plant-based meat alternatives are products designed to imitate the appearance and experience of eating animal meat This category can include ‘mock meats’—meat analogues made from soy or gluten [27] Examples: Impossible Foods, Beyond Meat and mock chicken
**Insect Protein**	Traditional part of diets in many African, South American and Asian countries	Edible insects that are cooked prior to consumption or eaten raw Examples: whole insects such as crickets	The first bread using insect flour (Fazer Sirkkaleipä or Fazer Cricket Bread) was created in 2017 by Finnish company, Fazer	Processed edible insects usually added as ingredients in processed food products Examples: bread or biscuits made with cricket flour; insect burger patties
**Algal Protein**	Traditional part of diets, especially in Asian countries	Edible algae that are typically processed or cooked prior to consumption Examples: seaweed, *Spirulina* and *Chlorella*	Recent decades have seen a rise in nutraceuticals and functional foods incorporated with algae or its extracted ingredients [24,28]	Processed edible algae which can be consumed whole, used as ingredients in processed food products or their components (such as bioactive compounds) extracted and used in health food products Examples: foods incorporated with seaweed, *Spirulina,* or *Chlorella*, such as pasta, beverages, and desserts
**In vitro/Cultured Meat Protein**	NA	NA	The first in vitro meat patty was created in 2013. It was cultivated using stem cells extracted from the biopsy of a cow.	Laboratory-grown meat using cell-based technologies to culture and grow animal cells Examples: Laboratory-grown beef burger patties, beef meatballs, and chicken tenders

**Table 2:** Consumer perceptions and available evidence on the nutritional quality of alternative proteins.

	Consumer Perceptions	Nutritional Evidence
**Plants**	Plant-based diets are generally perceived as healthy [33] Plant-based meat alternatives (e.g., Impossible Foods, Beyond Meat): Consumer perceptions are not well studied Certain consumer groups are more ready to accept them e.g., more affluent Asian populations [13]	Plant-based diets: Clinical trials have found favourable changes in cardiovascular biomarkers [43] Longitudinal studies have found lower risks of chronic diseases and mortality [44] Plant-based meat alternatives: Generally similar nutritionally to conventional meat though lower in protein and higher in sodium (Tables 3–6) Limited studies, especially randomised controlled trials or longitudinal studies
**Insects**	Perceived as nutritious and high in protein, though more disgusting or dangerous [42,45]	High in protein, fibre, and minerals [46] However, this is variable within and between species Effects on health are not well studied
**Algae**	Perceived as having functional benefits [22,47]	High protein content (comparable to soybean), contains EPA ^1^, DHA ^2^, bioactive compounds and antioxidants [28] However, this is variable within and between species, and depends on location and season Not well studied in humans though in vitro and animal studies have demonstrated antioxidant, anti-inflammatory and other possible health benefits [48]
**Cultured meat**	Consumer perceptions are not well studied Certain consumer groups are more ready to accept them e.g., more affluent Asian populations [13] Some European consumers are uncertain about the health benefits of cultured meat [26]	Not commercially available No available clinical evidence from randomised controlled trials

^1^ EPA: Eicosapentaenoic acid; ^2^ DHA: Docosahexaenoic acid.

**Table 3:** Comparison of conventional meat, processed fish sources and alternative protein (mock chicken), as prepared and ready-to-eat.

	Beef Patty (Grilled) ^1^		Fish (Steamed) ^1^		Fishballs (Boiled) ^2^		Fishballs (Deep-Fried) ^2^		Chicken Breast (Grilled) ^1^		Mock Chicken (Stir-Fried) ^3^	
	Per Serving (120 g)	Per 100 g	Per Serving (113 g)	Per 100 g	Per Serving (74 g)	Per 100 g	Per Serving (82 g)	Per 100 g	Per Serving (120 g)	Per 100 g	Per Serving	Per 100 g
Calories (kcal)	326.40	272.00	181.00	160.00	49.00	66.21	71.09	86.70	247.20	206.00	106.00	110.7
Protein (g)	30.54	25.45	29.10	25.80	7.32	9.90	11.64	14.20	30.86	25.72	11.00	11.49
Carbohydrate (g)	0	0	0	0	3.39	4.60	1.15	1.40	0	0	6.00	6.27
Sugar (g)	0	0	0	0	0.72	0.99	0.25	0.30	0	0	3.00	3.13
Total Fat (g)	21.82	18.18	6.25	5.53	0.66	0.90	2.21	2.70	12.74	10.62	4.25	4.44
Saturated Fat (g)	8.34	6.95	1.15	1.02	0	0.001	1.39	1.70	3.02	2.52	0.17	0.17
Polyunsaturated Fat (g)	0.62	0.51	1.15	1.02	-	-	0.16	0.20	3.01	2.51	-	-
Monounsaturated Fat (g)	9.83	8.19	1.92	1.70	-	-	0.57	0.70	4.83	4.02	-	-
Cholesterol (mg)	104.40	87.00	65.50	58.00	23.67	32.00	9.02	11.00	114.00	95.00	0	0
Sodium (mg)	459.60	383.00	460.00	407.00	486.99	658.00	533.00	650.00	394.80	329.00	873.00	911.75
Fibre (g)	0	0	0	0	0.06	0.10	0	0	0	0	1.00	1.04
Vitamin B12 (µg)	3.30	2.75	5.02	4.44	-	-	0	-	0.20	0.17	-	-
Iron (mg)	2.89	2.41	0.54	0.48	0.36	0.50	2.38	2.90	0.64	0.53	-	-
Zinc (mg)	7.40	6.17	0.55	0.49	-	-	0.25	0.30	1.04	0.87	-	-
Protein Source	Beef		Fish		Combination of fish meat				Chicken		Gluten	

Sources: ^1^ United States Department of Agriculture [52], ^2^ Health Promotion Board [53], ^3^ On-pack nutritional label. Nutritional information of all food items (as prepared and ready-to-eat) was calculated by adding fat (oil or butter) and sodium (salt or soya sauce) to raw ingredients. Dashes denote unavailable values.

**Table 4:** Comparison of beef and alternative proteins (legumes, algae, and insects), as prepared and ready-to-eat.

	Beef Patty (Grilled) ^1^		Silken Tofu (Boiled) ^1^		Tofu (Fried) ^1^		Tempeh, (Fried) ^1^		Black Beans (Boiled) ^1^		Seaweed (*Spirulina*) Salad (with Seasoning) ^1^		Bread with Cricket Flour (with Butter) ^2^		White Bread (with Butter) ^1^	
	Per Serving (120 g)	Per 100 g	Per Serving (60 g)	Per 100 g	Per Serving (93 g)	Per 100 g	Per Serving (90 g)	Per 100 g	Per Serving (120 g)	Per 100 g	Per Serving (80 g)	Per 100 g	Per Serving (80 g or 2 Slices)	Per 100 g	Per Serving (80 g or 2 Slices)	Per 100 g
Calories (kcal)	326.40	272.00	39.60	43.95	84.60	91.71	206.22	229.14	158.40	132.00	36.02	45.02	327.26	347.41	297.20	315.50
Protein (g)	30.54	25.45	4.00	4.44	4.00	4.34	18.04	20.04	10.63	8.86	4.52	5.65	21.63	22.96	5.46	5.79
Carbohydrate (g)	0	0	1.00	1.11	1.00	1.08	6.79	7.55	28.45	23.71	2.28	2.85	-	-	33.79	35.87
Sugar (g)	0	0	0	0	0	0	-	-	0.38	0.32	0.81	1.01	-	-	3.56	3.78
Total Fat (g)	21.82	18.18	2.00	2.22	6.50	7.05	13.60	15.11	0.65	0.54	1.83	2.29	-	-	15.05	15.98
Saturated Fat (g)	8.34	6.95	0	0	0.33	0.36	2.55	2.83	0.17	0.14	0.21	0.26	-	-	7.29	7.74
Polyunsaturated Fat (g)	0.62	0.51	1.00	1.11	2.27	2.46	4.95	5.5	0.28	0.23	0.51	0.64	-	-	-	-
Monounsaturated Fat (g)	9.83	8.19	0	0	3.35	3.63	5.38	5.98	0.06	0.05	1.01	1.26	-	-	-	-
Cholesterol (mg)	104.40	87.00	0	0	0	0	0	0	0	0	0	0	-	-	30.50	32.38
Sodium (mg)	459.60	383.00	333.00	369.59	514.60	557.83	452.44	502.72	284.40	237.00	567.72	709.65	-	-	340.10	361.04
Fibre (g)	0	0	0	0	0	0	-	-	10.44	8.70	0.28	0.35	1.95	2.07	1.76	1.87
Vitamin B12 (µg)	3.30	2.75	-	-	-	-	0.07	0.08	0	0	-	-	-	-	-	-
Iron (mg)	2.89	2.41	0.72	0.80	0.72	0.78	2.40	2.67	2.52	2.10	2.05	2.56	-	-	1.92	2.04
Zinc (mg)	7.40	6.17	-	-	-	-	1.01	1.13	1.34	1.12	0.16	0.20	-	-	-	-
Protein Source	Beef		Soy		Soy		Soy		Black beans		Algae (*Spirulina*)		Crickets		Wheat	

Sources: ^1^ United States Department of Agriculture [52], ^2^ Osimani et al. 2018 [54]. Nutritional information of all food items (as prepared and ready-to-eat) was calculated by adding fat (oil or butter) and sodium (salt or soya sauce) to raw ingredients. Dashes denote unavailable values.

**Table 5:** Comparison of animal- and plant-based burger patties (grilled).

	Beef Patty ^1^		Impossible Burger Patty ^2^		Beyond Burger Patty ^3^		Quorn Burger Patty ^3^		Insect Burger Patty ^2^		Black Bean Patty ^1^	
	Per Serving (120 g)	Per 100 g	Per Serving (113 g)	Per 100 g	Per Serving (113 g)	Per 100 g	Per Serving (80 g)	Per 100 g	Per Serving (98 g)	Per 100 g	Per Serving (71 g)	Per 100 g
Calories (kcal)	326.40	272.00	260.00	228.57	280.00	246.15	158.18	195.89	293.76	299.75	160.00	223.00
Protein (g)	30.54	25.45	19.00	16.70	20.00	17.58	12.55	15.54	20.23	20.65	6.00	8.36
Carbohydrate (g)	0	0	9.00	7.91	5.00	4.40	5.82	7.21	4.38	4.47	15.00	20.91
Sugar (g)	0	0	1.00	0.88	0	0	0.55	0.68	1.36	1.39	3.00	4.18
Total Fat (g)	21.82	18.18	16.25	14.29	20.25	17.80	8.61	10.67	20.67	21.09	9.25	12.89
Saturated Fat (g)	8.34	6.95	8.17	7.18	5.17	4.54	2.89	3.58	2.20	2.25	0.66	0.92
Polyunsaturated Fat (g)	0.62	0.51	-	-	-	-	-	-	-	-	-	-
Monounsaturated Fat (g)	9.83	8.19	-	-	-	-	-	-	-	-	-	-
Cholesterol (mg)	104.40	87.00	0	0	0	0	-	-	-	-	0	0
Sodium (mg)	459.60	383.00	870.00	764.84	850.00	747.25	863.64	1069.52	1108.88	1131.51	820.00	1142.86
Fibre (g)	0	0	3.00	2.64	2.00	1.76	3.64	4.50	-	-	2.98	4.15
Vitamin B12 (µg)	3.30	2.75	-	-	-	-	-	-	-	-	-	-
Iron (mg)	2.89	2.41	4.20	3.69	4.00	3.52	-	-	-	-	1.20	1.67
Zinc (mg)	7.40	6.17	-	-	-	-	-	-	-	-	-	-
Protein Source	Beef		Soy		Pea protein, rice protein, mung bean protein		Mycoprotein, fermented from the fungus *Fusarium venenatum*		Buffalo worms, soy		Black beans, brown rice	

Sources: ^1^ United States Department of Agriculture [52], ^2^ Internet sources [55,56], ^3^ On-pack nutritional labels. Nutritional information of all food items (as prepared and ready-to-eat) was calculated by adding fat (oil or butter) and sodium (salt or soya sauce) to raw ingredients. Dashes denote unavailable values.

**Table 7:** Food safety of alternative proteins.

Alternative Protein	Food Safety Aspects
**Plants**	Plants are generally safe to consume, though this depends on preparation and cooking techniques. Food safety of PBMAs is expected to be high due to controlled development and production conditions.
**Insects**	If improperly cleaned or processed, can be hosts or vectors for parasites; may increase risks of allergy and other health problems [20,152]. May pose less risk of transmitting zoonotic diseases to humans compared to animals and birds [156].
**Algae**	If improperly cleaned or processed, may accumulate heavy metals and iodine. Risk of containing toxins, allergens, pathogens, and pesticides. However, these risks are dependent on habitat [157].
**Cultured Meat**	Unknown, as not commercially available. Research suggests less concerns and greater acceptance of ‘hybrid meat’ products rather than cultured meat on their own.

## Data Availability

Data presented are from publicly available online databases: the United States Department of Agriculture’s FoodData Central database [52] and Singapore’s Health Promotion Board’s Energy & Nutrient Composition of Food database [53].
